# LONG-TERM PAID CARE WORKFORCE: SHORTAGE, BURNOUT, TRAINING, TURNOVER, WHAT’S NEXT?

**DOI:** 10.1093/geroni/igad104.1066

**Published:** 2023-12-21

**Authors:** Ann Rhodes, Anne Martin-Matthews

## Abstract

Although the long-term care workforce has struggled historically with staff satisfaction, safety, and retention, the pandemic triggered increased turnover and higher levels of staff burnout. As a result, many residential care organizations are using agency staff at unprecedented levels. Given that continuity of care is central to high-quality person-centered care, and the workforce should have access to a physically and psychologically safe environment, it is imperative that the gerontological research and practical community understand the changing conditions experienced by this workforce in crisis and offers solutions at the workplace, industry, and policy levels. In this symposium, authors explore different facets of paid long-term care workforce to stimulate thought and discussion. One paper contrasts the well-being of the workforce to that of the residents during COVID-19. A second paper presents workforce management capabilities and strategies and posits essential questions for the capacity of management and leadership needs of the workforce. Two papers highlight the struggles that are presented as paid long-term care relies increasingly on contract, short-term workers. Paper three discusses how the use of the contract workforce does not support the provision of person-centered care for conditions like dementia. Paper four uses payroll-based journal data to delve into short and long-term use of contract workforce and discusses the nature of contract staffing, and considers how contract staffing may be disruptive to both the direct care worker and the nursing home environment. The discussant will draw out the policy and practice implications arising from the papers. This is a collaborative symposium between the Aging Workforce and Paid Caregiving Interest Groups.

